# Rising surface salinity and declining sea ice: A new Southern Ocean state revealed by satellites

**DOI:** 10.1073/pnas.2500440122

**Published:** 2025-06-30

**Authors:** Alessandro Silvano, Aditya Narayanan, Rafael Catany, Estrella Olmedo, Verónica González‐Gambau, Antonio Turiel, Roberto Sabia, Matthew R. Mazloff, Theo Spira, F. Alexander Haumann, Alberto C. Naveira Garabato

**Affiliations:** ^a^Ocean and Earth Science, National Oceanography Centre, University of Southampton, Southampton SO14 3ZH, United Kingdom; ^b^ARGANS Ltd., Plymouth PL6 8BX, United Kingdom; ^c^Albavalor, Sociedad Limitada, Calle Catedrático Agustín Escardino, 9, Paterna Valencia 46980, Spain; ^d^Barcelona Expert Center on Remote Sensing, Institut de Ciències del Mar, Consejo Superior de Investigaciones Científicas, Barcelona 08003, Spain; ^e^European Space Agency, European Space Research Institute, Frascati 00044, Italy; ^f^Scripps Institution of Oceanography, University of California, San Diego, La Jolla, CA 92093-0202; ^g^Department of Marine Sciences, University of Gothenburg, Gothenburg 405 30, Sweden; ^h^Alfred Wegener Institute, Helmholtz Centre for Polar and Marine Research, Bremerhaven 27570, Germany; ^i^Department of Geography, Ludwig Maximilian University of Munich, Munich 80333, Germany

**Keywords:** sea ice, Antarctica, ocean warming, ocean salinity, satellites

## Abstract

For decades, the surface of the polar Southern Ocean (south of 50°S) has been freshening—an expected response to a warming climate. This freshening enhanced upper-ocean stratification, reducing the upward transport of subsurface heat and possibly contributing to sea ice expansion. It also limited the formation of open-ocean polynyas. Using satellite observations, we reveal a marked increase in surface salinity across the circumpolar Southern Ocean since 2015. This shift has weakened upper-ocean stratification, coinciding with a dramatic decline in Antarctic sea ice coverage. Additionally, rising salinity facilitated the reemergence of the Maud Rise polynya in the Weddell Sea, a phenomenon last observed in the mid-1970s. Crucially, we demonstrate that satellites can now monitor these changes in real time, providing essential evidence of the Southern Ocean’s potential transition toward persistently reduced sea ice coverage.

The surface of the polar Southern Ocean has been freshening since the early 1980s (1), coinciding with an expansion of Antarctic sea ice (2). However, this trend reversed abruptly after 2015, coinciding with a record-low sea ice extent in late 2016 (Fig. 1*A*). Since then, sea ice has remained at low levels, with multiple record minima in both summer and winter (2). Moreover, during the period of extensive sea ice coverage, large open-ocean polynyas were absent, but they reemerged over Maud Rise in the Weddell Sea in 2016 and 2017 (3).

**Fig. 1.**
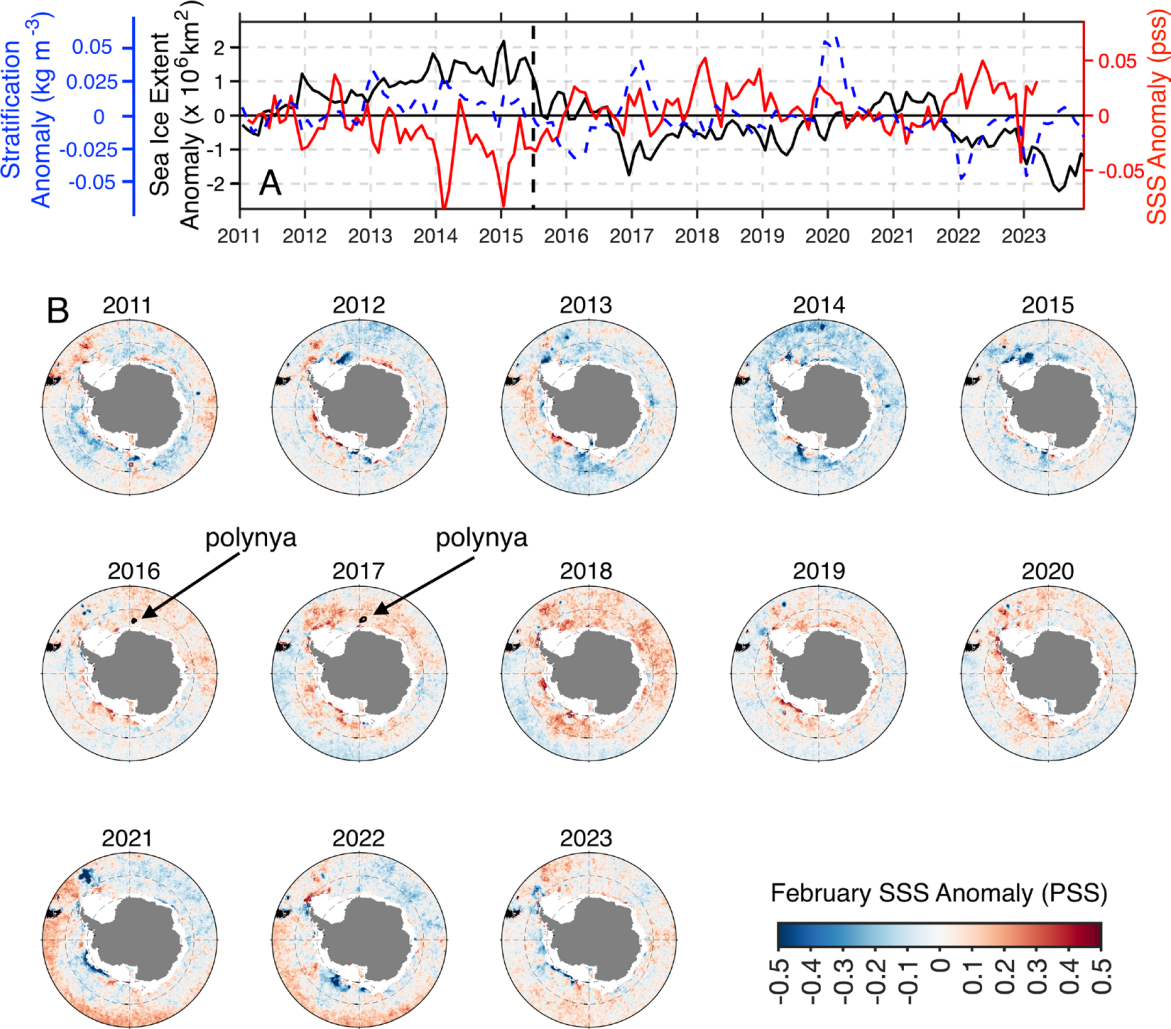
(*A*) Satellite-derived SSS in red (dimensionless, pss) and Antarctic sea ice extent in black (millions of km^2^). SSS is spatially averaged over the ice-free polar Southern Ocean (south of 50°S). Stratification anomalies are in dashed blue (kg m^−3^). Here, stratification is defined as the potential density difference between 200 m depth and the surface, estimated from Argo floats. The vertical dashed black line marks winter 2015, when sea ice retreat began. (*B*) Satellite-derived maps of February SSS anomaly. Summer values are shown at the minimum sea ice cover, when satellite retrieval can capture surface properties over most of the polar Southern Ocean. The area of winter polynyas (3) in the eastern Weddell Sea are highlighted in 2016 and 2017. Monthly anomalies are estimated by removing the mean seasonal cycle.

Several hypotheses have been proposed to explain the sea ice retreat, including changing atmospheric heat advection and wind patterns as well as upper-ocean warming (Fig. 2*B*; 2, 4). Two recent studies (2, 5) identified increased spatial (i.e., circumpolar) coherence, variance, and persistence of Antarctic sea ice anomalies, indicative of an abrupt critical transition in the system and a possible new state. While this critical transition has been put forward based on dynamical systems theory (5), the underpinning physical mechanisms remain unclear, limiting our ability to fully assess whether a regime shift has already occurred.

**Fig. 2.**
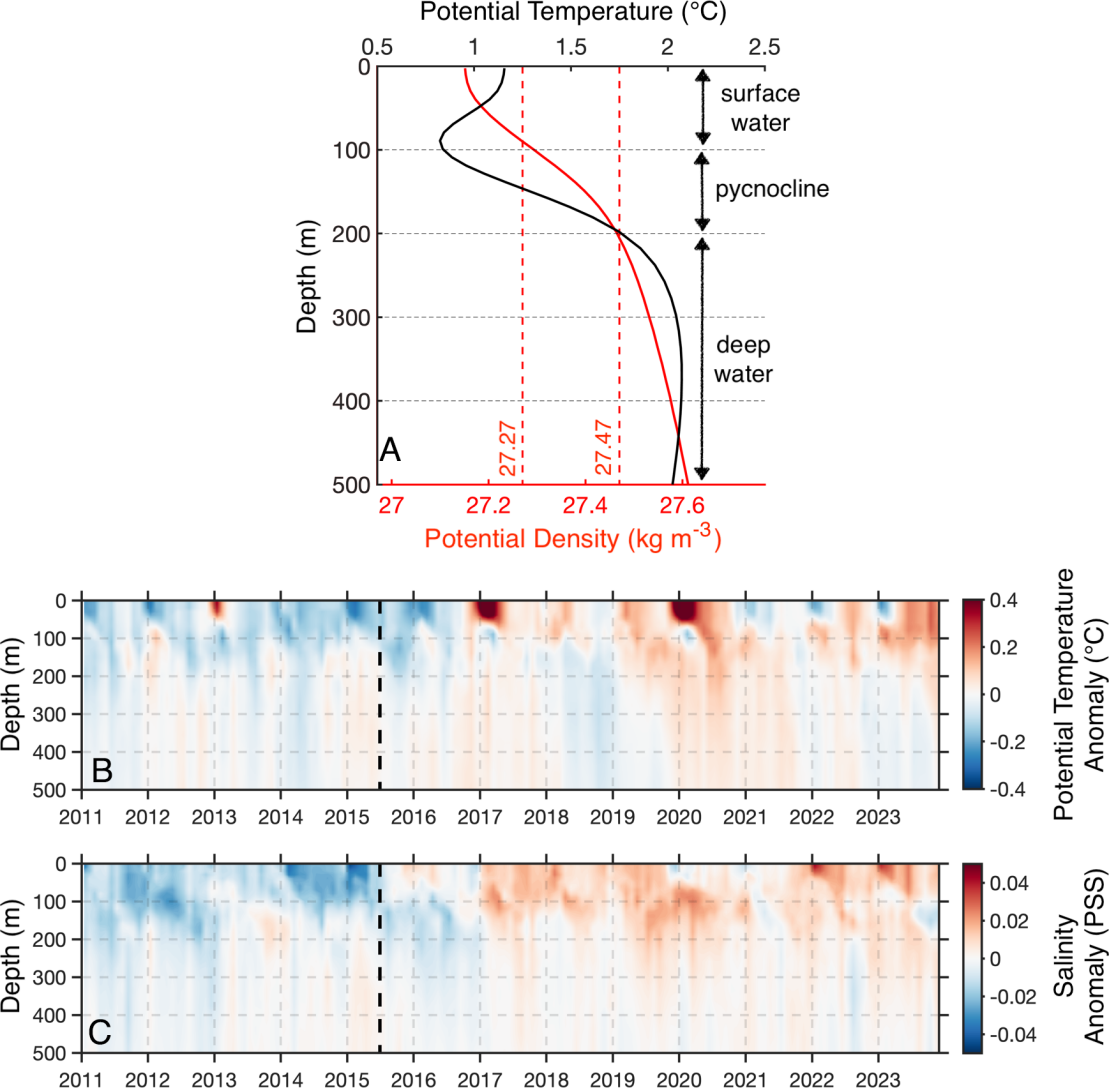
(*A*) Mean vertical profile of potential temperature (°C, black) and potential density referenced to the surface (kg m^−3^, red) from Argo float observations. Salinity follows the same structure as potential density. (*B*) Argo-derived potential temperature anomaly (°C) in the top 500 m of the water column. The vertical dashed black line marks winter 2015, when sea ice retreat began. (*C*) Same as (*B*), but for salinity. Argo-based observations are spatially averaged between 55°S and 65°S to capture the seasonally ice-covered Southern Ocean. Panel (*A*) also applies a time average over the 2011–2023 period. Monthly anomalies are calculated by removing the mean seasonal cycle.

Atmospheric processes alone seem insufficient to explain the abrupt sea ice retreat in 2015–16 and its subsequent multiyear decline (5), underscoring the critical role of oceanic processes and ice–ocean feedback (2, 6, 7). Sea ice changes are closely linked to upper-ocean stratification which, at these latitudes, is primarily controlled by salinity. In the polar Southern Ocean, cold, fresh surface waters overlay warmer, saltier deep waters (Fig. 2*A*). During winter, surface cooling and sea ice formation reduce stratification, allowing vertical mixing to transport heat upward, either melting sea ice from below or limiting its growth (8). However, decades of surface freshening strengthened stratification, trapping subsurface heat at depth, sustaining expanded sea ice coverage (7, 9) and limiting deep convection along with open-ocean polynyas (10). Here, we show that since 2015, these conditions have reversed: Surface salinity in the polar Southern Ocean has increased, upper-ocean stratification has weakened, sea ice has reached multiple record lows, and open-ocean polynyas have reemerged.

## Results and Discussion

In this study, we combine a new satellite-derived regional product of sea surface salinity (SSS), satellite-derived sea ice extent, and in situ hydrographic profiles from Argo floats (*SI Appendix*, *Extended Methods*) to assess upper-ocean changes associated with the recent decline in Antarctic sea ice. In situ measurements indicate a fresher upper ocean before mid-2015, followed by a period of salinification, with the strongest salinity anomalies observed in the top 100 to 200 m of the water column (Fig. 2*C*), extending thus down to the pycnocline that separates cold, fresh surface waters from warmer, saltier deep waters (Fig. 2*A*). Satellite-derived SSS effectively captures the abrupt increase in 2015–2016 and the sustained high values that followed (Fig. 1*A*). The correlation between satellite-derived SSS and sea ice extent is strong and negative (R = −0.62, 95% significance), further reinforcing their connection. This salinity pattern is observed circumpolarly, with anomalies exceeding 0.2 pss in some locations (Fig. 1*B*).

Increased SSS is associated with reduced upper-ocean stratification (see the blue dashed line in Fig. 1*A*). Consistent with salinity trends, stratification declined in 2015–2016 and has remained low since. Exceptions occurred during the austral summers of 2016–2017 and 2019–2020, when abrupt surface warming driven by wind anomalies temporarily enhanced stratification (11). Notably, previous studies (1, 7, 9) show that the opposite pattern—greater upper-ocean stratification and surface freshening—characterized periods of sea ice expansion, including up to 2014. Our findings align with recent modeling work (6, 12), showing that reduced stratification and increased surface salinity are contributing to Antarctic sea ice retreat. Finally, the eastern Weddell Sea experienced enhanced surface salinity in years of polynya openings (Fig. 1*B*), weakening stratification and favoring the reemergence of the Maud Rise polynya (3). This contrasts with previous decades, which saw freshening and an absence of large polynyas.

Our work identifies surface salinity in the polar Southern Ocean as a fingerprint of upper-ocean stratification, which ultimately regulates Antarctic sea ice cover and open-ocean polynyas. Crucially, we demonstrate that this salinity signature can be monitored via satellites. Sustained satellite observations of surface salinity will thus be essential for determining whether Antarctic sea ice is undergoing a long-term shift toward persistently low coverage. Anthropogenic forcing is generally expected to drive surface freshening and increased stratification in the polar oceans. Indeed, modeling studies (13, 14) predict freshening in the Southern Ocean due to intensified equatorward transport of fresh polar waters, enhanced precipitation, and increased Antarctic Ice Sheet melting. However, the rapid changes observed over the past decade 1) contradict the prevailing expectation of anthropogenic-driven freshening and 2) are unprecedented in the satellite record. This suggests that current understanding and observations may be insufficient to accurately predict future changes. Continuous satellite missions and in situ monitoring are now more critical than ever to track and understand the drivers of recent and future shifts in the ice–ocean system, including atmospheric forcing, ocean dynamics, and ice–ocean–atmosphere feedbacks.

## Materials and Methods

The SSS product consists of a 2011–2023 time series of 9-d Level 3 maps generated daily at 25 km resolution over the Southern Ocean (30°S to 90°S) using the Soil Moisture and Ocean Salinity satellite. Sea ice extent is derived by satellites (15). In situ data are from the Argo programme. See *SI Appendix*, *Extended Methods* for data access and further information.

## Supplementary Material

Appendix 01 (PDF)

## Data Availability

Data from argo floats are deposited in https://sio-argo.ucsd.edu/RG_Climatology.html (16); satellite-derived Sea Surface Salinity (SSS) data can be found at https://opensciencedata.esa.int/products/sofresh-sea-surface-salinity/collection (17); Sea ice extent data are from OSI SAF (https://osisaf-hl.met.no/v2p2-sea-ice-index) (15). All other data are included in the manuscript and/or *SI Appendix*.

## References

[1] F. Haumann, N. Gruber, M. Münnich, I. Frenger, S. Kern, Sea-ice transport driving Southern Ocean salinity and its recent trends. Nature **537**, 89–92 (2016). 27582222 10.1038/nature19101

[2] A. Purich, E. W. Doddridge, Record low Antarctic sea ice coverage indicates a new sea ice state. Commun. Earth Environ. **4**, 314 (2023).

[3] E. C. Campbell , Antarctic offshore polynyas linked to Southern Hemisphere climate anomalies. Nature **570**, 319–325 (2019). 31182856 10.1038/s41586-019-1294-0

[4] C. Eayrs, X. Li, M. N. Raphael, D. M. Holland, Rapid decline in Antarctic sea ice in recent years hints at future change. Nat. Geosci. **14**, 460–464 (2021).

[5] W. Hobbs , Observational evidence for a regime shift in summer Antarctic sea ice. J. Clim. **35**, 2263–2275 (2024).

[6] L. T. Zhang , The relative role of the subsurface Southern Ocean in driving negative Antarctic sea ice extent anomalies in 2016–2021. Commun. Earth Environ. **3**, 302 (2022).

[7] H. Goosse, V. Zunz, Decadal trends in the Antarctic sea ice extent ultimately controlled by ice–ocean feedback. Cryosphere **8**, 453–470 (2014).

[8] D. G. Martinson, Evolution of the Southern Ocean winter mixed layer and sea ice: Open ocean deepwater formation and ventilation. J. Geophys. Res. **95**, 11641–11654 (1990).

[9] O. Lecomte , Vertical ocean heat redistribution sustaining sea-ice concentration trends in the Ross Sea. Nat. Commun. **8**, 258 (2017). 28811497 10.1038/s41467-017-00347-4 PMC5557847

[10] C. de Lavergne, J. Palter, E. Galbraith, R. Bernardello, I. Marinov, Cessation of deep convection in the open Southern Ocean under anthropogenic climate change. Nat. Clim. Change **4**, 278–282 (2014).

[11] E. A. Wilson, D. B. Bonan, A. F. Thompson, N. Armstrong, S. C. Riser, Mechanisms for abrupt summertime circumpolar surface warming in the Southern Ocean. J. Clim. **1**, 1–36 (2023).

[12] Y. Morioka , Antarctic sea ice multidecadal variability triggered by Southern Annular Mode and deep convection. Commun. Earth Environ. **5**, 633 (2024).

[13] N. C. Swart, S. T. Gille, J. C. Fyfe, N. P. Gillett, Recent Southern Ocean warming and freshening driven by greenhouse gas emissions and ozone depletion. Nat. Geosci. **11**, 836–841 (2018).

[14] R. Bintanja, G. J. van Oldenborgh, S. S. Drijfhout, B. Wouters, C. A. Katsman, Important role for ocean warming and increased ice-shelf melt in Antarctic sea-ice expansion. Nat. Geosci. **6**, 376–379 (2013).

[15] OSI SAF Sea ice index 1978-onwards, version 2.2, OSI-420. EUMETSAT Ocean and Sea Ice Satellite Application Facility. https://osisaf-hl.met.no/v2p2-sea-ice-index. Accessed 23 August 2024.

[16] D. Roemmich, J. Gilson, The 2004–2008 mean and annual cycle of temperature, salinity, and steric height in the global ocean from the Argo program. Prog. Oceanogr. **82**, 81–100 (2009).

[17] V. González-Gambau, E. Olmedo, A. García-Espriu, C. González-Haro, A. Turiel, Southern Ocean Sea Surface Salinity Level 3 maps. DIGITAL.CSIC. 10.20350/digitalCSIC/15493. Deposited 31 July 2023.

