# Supplementary material for: Rising surface salinity and declining sea ice: A new Southern Ocean state revealed by satellites
Source: Proc Natl Acad Sci U S A. 2025 Jun 30;122(27):e2500440122. doi: 10.1073/pnas.2500440122 (PMC12260588; doi:10.1073/pnas.2500440122)
Supplement: Supplementary file 1 — Appendix 01 (PDF) [file pnas.2500440122.sapp.pdf]

Extended Methods

Satellite-derived Sea Surface Salinity

The dedicated Southern Ocean SSS product has been developed from ESA's Soil Moisture and Ocean Salinity (SMOS) data by the Barcelona Expert Center (BEC, http://bec.icm.csic.es). The spatial resolution of salinity retrieval from a SMOS overpass is approximately 50 km (1). The regional product in the Southern Ocean consists of a 2011–2023 time series of 9-day Level 3 salinity maps generated daily at an EASE-SL 25 km grid covering the region in 30ºS-90ºS (2). Data are available between February 2011 and March 2023. The retrieval of satellite SSS in polar regions is a grand challenge due to several technical difficulties, such as the contamination of the radiometric signal close to sea ice (strong biases that appear near the ice-edge), the low signal-to-noise ratio of SSS due to the reduced sensitivity of L-band radiometry to salinity in cold waters (3) and the reduced variability of SSS in the Southern Ocean (unlike the SSS variability in the Arctic Ocean).

Dedicated algorithms have been tailored for the mitigation of the sea ice contamination and improving the signal-to-noise ratio. First, a sea ice mask was introduced when applying the Nodal Sampling technique to reduce the noise in the SSS retrievals (4, 5). Second, the debiased non-Bayesian retrieval scheme was applied to characterise and mitigate spatial biases in SSS depending on the acquisition conditions (6) and, for the first time, as a function of the distance to the ice edge. Finally, we applied a sea-ice mask that removes all the salinity values closer than 100 km to the ice edge and coast. Using different distances (e.g. 200 km) did not affect the results of the analyses. In these three steps, the sea-ice mask was based on the sea-ice concentration product provided by OSISAF (7). An extensive validation was performed by reference to in-situ salinity measurements from marine mammals (8), thermosalinograph data from research vessels (9), and data from Vendée Globe and Barcelona World Races (10, 11), as well as other in-situ databases available at Salinity Pilot Mission Exploitation Platform - Pi-MEP (https://www.salinity-pimep.org/reports/mdb.html). The comparison of the satellite data with the in-situ measurements showed almost zero bias and a standard deviation of the difference of 0.17 pss (compared to Argo), 0.22 pss (compared to marine mammals) and 0.25 pss (compared to thermosalinograph data from research vessels) for areas at least 100 km from the ice edge and coast. Note that the satellite and in-situ data provide measurements at different spatial and temporal scales (integrated area of 25 km^2 vs punctual acquisition, 9-day integrated vs instantaneous measurements), introducing therefore a representation or sampling-related error. Therefore, the standard deviation of the difference between satellite and in-situ data is not only due to the inaccuracy in the satellite product, but also due to the discrepancy in the represented scales. In particular, in more dynamic regions (such as areas closer to the ice-edge or coast), the sampling-related error is expected to be higher. The satellite product provided an adequate description of the Antarctic Circumpolar Current and seasonal and interannual variabilities consistent with the B-SOSE model (12). A complete description of the algorithms and the quality assessment are available online (13).

In-situ observations

In-situ temperature and salinity data are sourced from an Argo gridded (1° x 1°) product (14), which includes observations beneath sea ice. We use data from 2011 to 2023

to align with the satellite-derived SSS dataset. Stratification is defined as the potential density (referenced to the surface) difference between 200 m depth (the bottom of the pycnocline, see Fig. 2A) and the surface. No significant difference in time variability is observed when using alternative vertical ranges (e.g., 0-150m or 0-100m), nor when using different stratification metrics (e.g., Brunt–Väisälä frequency).

In Figs. 2 and 1A (stratification), data are averaged between 55°S and 65°S to capture the seasonally-ice covered Southern Ocean. We note that using a different region (e.g., between 50°S and 65°S) does not alter our results: salinification, warming, and reduced stratification in the upper ocean are consistently observed across the same period, in agreement with the SSS product. This in situ dataset has been shown to reliably capture the temporal variability of ocean properties on a circumpolar scale (15), demonstrating how Argo floats can monitor upper-ocean changes throughout all seasons.

Data, Materials, and Software Availability

Argo data: https://sio-argo.ucsd.edu/RG_Climatology.html; SSS data: http://bec.icm.csic.es/data-access-ftp/ ; https://opensciencedata.esa.int/products/sofresh-sea-surface-salinity/collection; Sea ice extent data are from OSI SAF: https://osisaf-hl.met.no/v2p2-sea-ice-index).

References

1. J. Font *et al.*, SMOS: the challenging sea surface salinity measurement from space, *Proc. IEEE*. **98**, 649 (2010).
2. V. González-Gambau, E. Olmedo, A. García-Espriu, C. González-Haro, A. Turiel, Southern Ocean Sea Surface Salinity Level 3 maps. https://doi.org/10.20350/digitalCSIC/15493 (2023).
3. S. H. Yueh, R. West, W. J. Wilson, F. K. Li, E. G. Njoku, Y. Rahmatsamii, Error sources and feasibility for microwave remote sensing of ocean surface salinity. *IEEE Trans. on Geosci. Rem. Sen.* **39**, 1049–1060 (2001).
4. V. González-Gambau, E. Olmedo, A. Turiel, J. Martínez, J. Ballabrera-Poy, M., Portabella, M. Piles, Enhancing SMOS brightness temperatures over the ocean using the nodal sampling image reconstruction technique, *Remote Sens. Environ.*, **180**, 205 – 220, https://doi.org/http://dx.doi.org/10.1016/j.rse.2015.12.032, http://www.sciencedirect.com/science/article/pii/S0034425715302534, special Issue: ESA’s Soil Moisture and Ocean Salinity Mission - Achievements and Applications (2016).
5. V. González-Gambau *et al.,* Benefits of Applying Nodal Sampling to SMOS Data Over Semi-Enclosed Seas and Strongly RFI-Contaminated Regions, in: IGARSS 2018 - 2018 IEEE International Geoscience and Remote Sensing Symposium, pp. 305–308, https://doi.org/10.1109/IGARSS.2018.8518510 (2018).
6. E. Olmedo, J. Martínez, A. Turiel, J. Ballabrera-Poy, M. Portabella, Debiased non-Bayesian retrieval: A novel approach to SMOS Sea Surface Salinity, *Remote Sens. Environ*. **193**, 103–126 (2017).
7. OSI SAF Global sea ice concentration interim climate data record (v3.0, 2022), OSI-430-a, doi:10.15770/EUM_SAF_OSI_0014. EUMETSAT Ocean and Sea Ice Satellite Application Facility.

8. F. Roquet *et al.*, MEOP-CTD in-situ data collection: a Southern ocean Marine-mammals calibrated sea water temperatures and salinities observations. Sea Scientific Open Data Publication. https://doi.org/10.17882/45461 (2024).
9. G. Aulicino, Y. Cotroneo, I. Ansorge, M. van den Berg, Sea surface temperature and salinity collected aboard the S.A. AGULHAS II and S.A. AGULHAS in the South Atlantic Ocean and Southern Ocean from 2010-12-08 to 2017-02-02 (NCEI Accession 0170743). NOAA National Centers for Environmental Information. Dataset. https://doi.org/10.7289/v56m3545 (2018).
10. M. Umbert, N. Hoareau, J. Salat, J. Salvador, Barcelona World Race 2014-2015 thermosalinograph data [Dataset]; DIGITAL.CSIC; https://doi.org/10.20350/digitalCSIC/16424; http://hdl.handle.net/10261/362395 (2023).
11. J. Salat, M. Umbert, J., Ballabrera-Poy, P. Fernández, J. Salvador, J. Martínez, The contribution of the Barcelona World Race to improve the ocean surface information. A validation of the SMOS remote sensed salinity. *IEEE Contributions to Science Institut d'Estudis Catalans* (2014).
12. A. Verdy, M. R. Mazloff, A data assimilating model for estimating Southern Ocean biogeochemistry. *J. Geophys. Res. Oceans* **122**, 6968–6988 (2017).
13. V. González-Gambau, E. Olmedo, A. García-Espriu, C. González-Haro, A. Turiel, BEC Southern Ocean L3 SSS Product Description. Technical note. Barcelona Expert Center. https://doi.org/10.20350/digitalCSIC/16664 (2024).
14. D. Roemmich, J. Gilson, The 2004–2008 mean and annual cycle of temperature, salinity, and steric height in the global ocean from the Argo program. *Prog. Oceanogr.* **82**, 81–100 (2009).
15. A. Purich, E. W. Doddridge, Record low Antarctic sea ice coverage indicates a new sea ice state. *Commun. Earth Environ.* **4**, 314 (2023).
